# High-resolution nuclear cell biology by cryo-electron tomography

**DOI:** 10.1080/19491034.2026.2675754

**Published:** 2026-06-01

**Authors:** Zanetta Kechagia, Ohad Medalia

**Affiliations:** Department of Biochemistry, University of Zurich, Zurich, Switzerland

**Keywords:** Cryo-ET, nucleus, nuclear lamina, nuclear pore complex, chromatin

## Abstract

The nucleus is a structurally diverse and dynamic organelle that anchors chromatin and orchestrates a large number of essential processes, including transcription, replication, ribosome biogenesis, and nucleocytoplasmic transport. Understanding how nuclear macromolecular assemblies are organized and coordinate these processes requires high-resolution imaging methods, capable of achieving sub-molecular resolution while preserving native cellular structures. Cryo-electron tomography (cryo-ET) now provides unprecedented three-dimensional views of nuclear architecture *in situ*, up to sub-nanometer resolution. In this review, we discuss how cryo-ET has reshaped our understanding of nuclear biology including chromatin organization, nuclear pore complex (NPC) architecture and dynamics, and chromatin – lamina interactions. We highlight how these insights have resolved long-standing debates in biology, linked nuclear structure to function, and set the stage for future developments that will bridge molecular and cellular scales.

## Introduction

The sheer concentration of proteins and nucleic acids in the cell nucleus [1] —exceeding 100 mg/ml – together with the dynamic properties of its macromolecular complexes, presents major challenges for resolving molecular interactions with a high spatial accuracy. Despite the crowded environment, the nucleus is a highly organized organelle whose architecture is hierarchically structured across multiple spatial and functional scales, with structure and function tightly interlinked. The packaging of meters-long DNA together with a vast array of regulatory proteins, RNA and cofactors, into a few micrometers compartment requires a remarkably ordered yet inherently dynamic organization [2]. This organization is further modulated by the physicochemical properties of the nuclear environment, which influence molecular interactions and higher-order architecture, to facilitate temporal and accurate cellular functions [3,4].

At the nuclear periphery, nuclear pore complexes (NPCs) are embedded in the nuclear envelope to mediate nucleocytoplasmic transport [5] while interacting with the nuclear lamina – a meshwork of lamin filaments and binding proteins [6,7]. The nuclear lamina components connect the cytoskeleton to chromatin through direct interactions, contributing to chromatin organization while simultaneously tuning the mechanical properties of the nucleus [8–10]. This multilevel structural continuum extends into the nuclear interior, where further nuclear machineries, such as the nucleolus, coordinate essential processes including ribosome biogenesis [2,4,11]. Consequently, even slight perturbations in this intricate organization can profoundly influence processes such as protein and gene expression, impacting cell behavior and fate. Understanding these multicomponent processes requires imaging modalities that can resolve molecular assemblies directly within their native context and with high spatial resolution.

**Figure 1.**
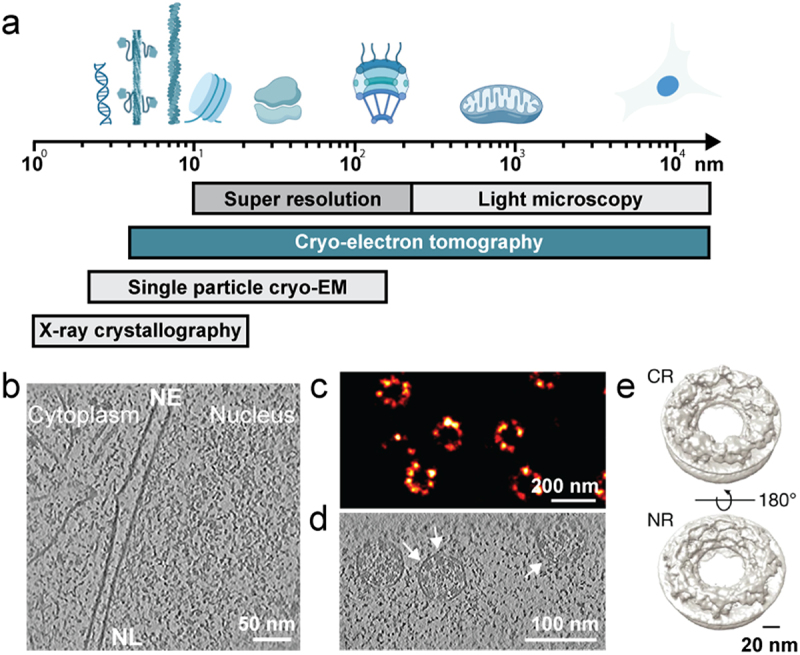
Bridging scales of resolution by combining variety of imaging modalities.

Microscopy has been instrumental in illuminating nuclear processes and their dynamics. However, the limited resolution of conventional light microscopy, and its reliance on fluorescent labels, restricts our ability to visualize the molecular landscape and to uncover functional interactions within the nucleus. Fluorescence microscopy enables live-cell imaging and has provided key insights into nuclear architecture and dynamics, but its resolution remains insufficient to resolve individual macromolecular assemblies (Figure 1a). Super-resolution fluorescence methods have extended the limits of localizing proteins to tens of nanometers (Figure 1a, c) yet provide limited contextual and structural information (Figure 1) [14]. Expansion microscopy further extends the resolution capabilities of light microscopy and provides an excellent means of localizing biological macromolecules [15]; however, its effective resolution depends on a combination of the physical expansion factor and diffraction-limited imaging, making it highly sensitive to sample preparation conditions and the preservation of structural integrity throughout the expansion process which is difficult to control reliably. Complementary approaches such as atomic force microscopy (AFM) can map the mechanical properties and topological landscape of nuclei or purified nuclear assemblies and capture time-dependent movements using high-speed AFM, but they too lack access to the internal molecular architecture of the intact nucleus [16,17].

a. Resolution ranges of major imaging techniques shown in nanometers. Approximate diameters of key nuclear components are indicated as examples (from the left DNA, lamin filament, actin filament, nucleosome, ribosome, nuclear pore complex, mitochondrion, and the nucleus).

b. Tomographic slice of a nuclear cross-section illustrating the nuclear periphery. From the left, the cytoplasm, nuclear envelope (NE), nuclear lamina (NL), and nuclear interior, where decorated nucleosome structures are clearly visible.

c. Representative 2D dSTORM image of U2OS cells expressing SNAP-tagged Nup96 [12]. Super resolution microscopy can be utilized for correlation with cryo-ET.

d. Example of a cryo-tomogram showing an x-y tomographic slice through the nuclear envelope, from human dermal fibroblast. The NPC inner ring, with individual subunits (white arrow) is seen in the reconstructed volume.

e. Three-dimensional average of an entire NPC from DLD-1 cells [13]. Nuclear ring (NR) and cytoplasmic ring (CR) indicating the nuclear and cytoplasmic face of the NPC.

**Figure 2.**
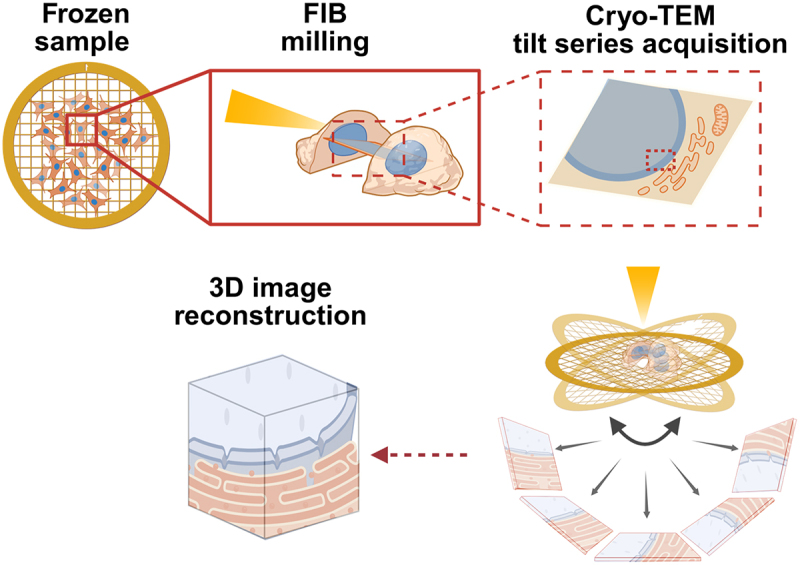
Workflow for cellular cryo-ET of nuclei.

Structural biology techniques such as X-ray crystallography, NMR spectroscopy, and single-particle cryo-electron microscopy (cryo-EM) have transformed the field of structural biology and thereby our understanding of molecular interactions [18–20] (Figure 1a), yet these methods typically rely on purified material. In contrast, cryo-electron tomography (cryo-ET) enables three-dimensional reconstructions of native cellular environments without averaging, providing direct visualization of macromolecular assemblies within intact vitrified cells (Figure 1a-b,d) [21]. When combined with rapid cryo-fixation and cryo-focused ion beam (cryo-FIB) milling to produce thin lamellae from otherwise inaccessible regions [22], cryo-ET can resolve cellular architecture at nanometer resolution, from the cytoplasm to the nucleus (Figures 1 and 2) [21,23]. Early applications of this technique laid the foundation for modern *in situ* structural biology [24] and helped resolve long-standing controversies and challenging classical models of nuclear organization [24,25]. In the sections that follow, we outline the principles of cryo-ET, highlight recent advances that have illuminated the molecular architecture and functional organization of the nucleus, and discuss the developments that are still required to push the field forward.

Vitrified cells are sectioned in the nuclear region using cryo-focused ion beam (cryo-FIB) milling to remove material above and below the area of interest, producing an electron-transparent lamella that can be transferred to the cryo-TEM for imaging. Tomographic data are acquired by tilting the sample typically between ±60°, while the electron beam targets the same region at each angle to generate a series of 2D projections. These projections are then computationally reconstructed into a 3D volume of the targeted nuclear area. Nuclei are marked blue.

## Visualizing the cellular landscape in 3D

The principle of cryo-ET has been discussed in a large number of reviews [21,23,26–28], therefore we will only briefly mention relevant key principles for its applications in eukaryotic cells.

Eukaryotic cells grown directly on or pipetted onto EM grids can be plunge-frozen in liquid nitrogen cooled ethane, producing vitreous ice that maintains the integrity and spatial organization of cells [29]. Larger specimens – such as small tissue fragments up to ~200 µm thick – require high-pressure freezing (HPF) to achieve vitrification [27,30,31]. HPF uses a rapid jet of liquid nitrogen under high pressure, thereby, suppressing ice crystal formation and preserving cellular and molecular structures. Although these approaches were developed several decades ago [32], further innovations are needed to ensure complete vitrification and to improve reproducibility as this is often a major bottleneck in cryo-EM workflows. For example, *C. elegans* worms and embryos are often not fully vitrified based on empirical data.

Following vitrification, samples can be studied by cryo-ET. However, most eukaryotic cells are too thick for direct imaging, as high-quality tomograms require specimens thinner than ~300 nm to favor predominantly single elastic scattered electrons, which is essential for acquiring interpretable structural information. Thicker samples have more frequent inelastic scattering, which reduces the signal-to-noise ratio and increases radiation damage [33]. Therefore, regions of interest must be physically thinned. Two main methods have been developed to achieve this goal: cryo-electron microscopy of vitrified sections (CEMOVIS), which produces thin cryosections using cryo-ultramicrotomy [34–36], and cryo-focused ion beam (cryo-FIB) milling, which is now the standard [37–39]. Cryo-FIB milling utilizes a focused beam of ions – generated from either a liquid metal ion source (LMIS) or most recently a plasma source [40]—to gradually remove material surrounding the region of interest (Figure 2). This process reliably produces thin lamellae, typically 150–200 nm thick, that serve as transparent windows into the cell interior and tissues [41,42]. Currently, cryo-FIB instruments can be equipped with a lift-out technology. These methodologies enable extraction of a vitrified block from a defined region of interest, followed by transfer of the block to another grid and serial sectioning to generate thin lamellae suitable for cryo-ET [43–46].

Thinned samples are then transferred to a Cryo-Transmission Electron Microscope (Cryo-TEM) for tomographic data acquisition. 3D information from a region of interest is obtained by acquiring a series of two-dimensional projection images, recorded from different angles by tilting the specimen around a single axis (Figure 2). These ‘tilt-series’ provide multiple views of the same region, which can then be computationally reconstructed into a 3D volume. Recent montage acquisition strategies further extend this approach by stitching adjacent tilt-series to increase the field of view, enabling imaging of larger continuous regions [47]. This can be particularly interesting for the study of nuclear structural organization, where larger regions of high-resolution detail are often required, for instance depicting chromatin organization. The introduction of direct electron detectors (DEDs) in cryo-EM marked a breakthrough for the field, as these cameras can track and correct sample motion during image acquisition. This capability enables high-resolution imaging while using low electron doses [48].

Despite these advances, several technical challenges remain. Image acquisition is limited by the tilt geometry of the sample and of the microscope, as samples can typically only be tilted up to ~±60° before they become too thick (Figure 2), leaving a portion of missing angular information – a limitation known as the ‘missing wedge’ [49]. This generates artificial elongation of structures in a direction parallel to the axis of the beam. Reducing the impact of the missing wedge effect and achieving a more isotropic resolution of tomograms remains an important aim [50]. Additionally, variations in ice thickness, lamella quality, and specimen charging often further degrade the image quality. Although the nucleus as a structure is easily identified, locating specific nuclear structures within large, feature-rich and low signal-to-noise cryo-tomograms presents an additional challenge [51].

While these challenges await further technological advances, the localization of specific components can already be addressed through correlative light and electron microscopy (cryo-CLEM), which enables fluorescence-guided targeting of regions of interest, and through the development of integrated cryo-fluorescence – cryo-EM platforms that minimize sample handling and transfer [52–54]. However, the identification of a single protein within the cellular context requires a labeling that can be recognized in cryo-tomograms [55]. Recently, a genetically targeted nanogold labeling strategy was introduced [56]. By delivering small, functionalized gold nanoparticles that covalently bind HaloTagged targets, the method provides molecular specificity, high EM contrast, and minimal perturbation, thereby expanding the range of proteins that can be identified within the native cellular environment. This is particularly relevant in the context of the nucleus where the identification of molecular targets in its dense environment is often challenging. On the computational side, advances in alignment algorithms, dose weighting, and deep-learning-based denoising of tomograms have improved the tomogram fidelity and contrast, while machine learning approaches increasingly help to mitigate the effects of the missing wedge [57–61]. More recently, integrated machine-learning workflows such as SPACEtomo enable end-to-end automation of cryo-ET acquisition by combining lamellae detection, biological feature segmentation and autonomous target selection, thereby increasing throughput and reducing operational bias [62].

Together, these developments have transformed cryo-ET into a mature approach capable of revealing architectural detail of cellular structures and processes, at nanometer resolution. In particular, the ability to image nuclear assemblies in situ and nuclear architecture has provided unprecedented insights into chromatin organization, nuclear pore complex architecture, and lamina – chromatin interactions that were previously inaccessible through traditional structural or imaging techniques.

## Unraveling the functional nuclear architecture by cryo-ET

The nucleus is a crowded compartment where structural complexity is intimately linked to highly regulated functions. Subtle changes in chromatin compaction, histone modifications, nuclear pore organization, or nuclear lamina architecture may influence transcriptional regulation and genome stability. Cryo-ET enables visualization of these structural alterations, bridging structural and cell biology, and therefore closing the gap between molecular-scale organization to cell-level behavior. Importantly, cryo-ET, unlike any other technique available, can visualize the entire environmental context of molecular interactions that surround a region of interest. This unique ability can offer novel, otherwise inaccessible and many times unexpected information. In the following sections we highlight some key examples where cryo-ET has redefined our understanding of nuclear architecture.

### Insight into chromatin architecture

The fundamental understanding of chromatin organization and dynamics remains one of the most important questions in cell biology, ever since it was first observed [63]. Classical models, derived from biochemical and *in vitro* imaging studies, suggested a hierarchical organization of nucleosomes into 10-nm ‘beads-on-a-string’ fiber, which can be further compacted into regular 30-nm fibers stabilized by the linker histone H1. Two main 30-nm fiber models – zigzag and solenoid – dominated structural interpretations for nearly half a century. However, direct visualization by cryo-ET of these structures in intact nuclei has consistently failed to confirm the presence of regular 30-nm fibers. These studies have provided conclusive evidence that chromatin organization in cells is irregular, heterogeneous and therefore, questioned the dominant chromatin organization models [64–68].

Using cryo-FIB milling followed by Cryo-ET and image processing, nucleosomes can be visualized and linker DNA can be often inferred computationally in their native nuclear environment. **Template matching** and neural network-based approaches allow to identify nucleosomes [9,67] that are then subjected to **Subtomogram averaging (STA)** [69]. Combining structural analysis with **back-mapping** of the nucleosome into the tomography volumes, in their native orientation, allows researchers to reconstruct consensus nucleosome structures and reinsert them into tomograms in their original orientation. Linker DNA trajectories can then be modeled using worm-like chain representations, revealing the three-dimensional geometry of chromatin fibers with sub-nanometer precision. The resolution of this approach, higher than 10 Å, is sensitive for small changes and variations in chromatin organization that previously could not be detected.

In human RPE-1 cells, chromatin packing appeared irregular and consistent with ‘liquid-like’ models of organization, lacking periodic 100–200 nm motifs or well-ordered fibers [70,71]. The most compact motif that was detected is the ordered stacked dinucleosome, while trinucleosome and tetranucleosome stacks as well as side-by-side dinucleosomes were absent from any well-defined structural classes, suggesting high variability in their stacking organization [67]. These observations support a flexible chromatin organization which can adopt a spectrum of conformations. On the other hand, in immature T lymphoblasts, heterochromatin near the nuclear periphery exhibited variable fiber widths (20–50 nm) composed of zigzag-like nucleosome arrays interspersed with linker-rich regions and nucleosome-free stretches [65]. Quantitative analyses revealed nucleosome spacings around 120 Å, less dense than expected from canonical fiber models, indicating flexible, context-dependent organization. Interestingly, at the nuclear periphery of resting T lymphocytes, a similar ~50-nm thick region of densely packed nucleosomes was observed [66]. STA of nucleosomes from this peripheral region revealed a characteristic orientation of H1-bound DNA around the nucleosome dyad. This conformation influences the inter-nucleosome linker-DNA trajectories. The study indicated that the local nucleosome concentrations varied widely (10–400 mg/mL), often exceeding 10 times the nuclear average DNA concentration (~10 mg/mL). Analyzing nucleosome pairs indicated short-range nucleosome contacts at 7 and 11 nm, but no long-range order, consistent with a liquid – gas intermediate state rather than a well packed 30 nm fiber [6].

These studies demonstrated how cryo-ET can be utilized to provide insights into the outstanding question of chromatin organization at the nuclear envelope. Moreover, they suggest that interphase chromatin organization is heterogeneous, flexible, and its density varies. Variability in linker length and nucleosome orientation can contribute to structural richness within chromosomes [65,72]. The structural differences observed across cell types and cell states further underscore the cell-specific nature of chromatin architecture [11]. Further analysis of molecular interactions, together with the identification of nucleosome variation classes and associated marks, may help rationalize these cell-type – specific structural differences. It is imperative to acknowledge that both T lymphocytes and RPE-1 cells have low levels of lamin A/C expression. Lamins, and their associated proteins, have a significant influence on the organization of chromatin at the nuclear envelope and it is widely recognized that this influence extends to the broader context of chromatin organization within the nucleus [11,73].

### Lamina–chromatin interactions

The nuclear lamina forms a structural and regulatory interface between chromatin and the nuclear envelope [74]. Composed mainly of A- and B-type lamins and their binding proteins, it provides mechanical support and anchors chromatin domains known as lamina-associated domains (LADs) [11,74]. Structural studies of the nuclear lamina are inherently challenging, owing to the need for high-resolution imaging to resolve its nanometer-scale architecture and the intrinsic difficulty of reconstituting well-defined lamin filaments in vitro. Cryo-electron tomography (cryo-ET) overcomes these limitations by enabling visualization of the lamina directly within its native cellular environment.

Using cryo-ET in detergent treated cells, it has been shown that both types of lamins assembled into thin filaments ~3.5 nm in diameter [75,76]. Functional differences between lamins were recently demonstrated, as lamin A/C and B-type lamins (B1/B2) were found to differentially influence peripheral chromatin density [9]. Depletion of A-type lamins reduces nucleosome density within ~100 nm of the lamina, while B-type lamin loss affects only the immediate periphery. This difference was explained structurally: lamin A contains a tail domain motif capable of directly binding nucleosomes, a feature absent in B-type lamins as well as in lamin C. The tail domain of lamin A is flexible, thus, direct interactions with chromatin may occur up to ~30 nm away from lamin filaments. However, interactions between the nuclear lamina and chromatin may also occur by a different set of proteins in cells e.g. LBR, LAP2 [73] or when different levels of lamin A are present.

These structural observations have important functional implications. Genome-wide analyses show that A-type lamin depletion leads to large-scale chromatin remodeling and transcriptional changes. B-type lamin depletion primarily alters nuclear morphology and envelope integrity but has less impact on chromatin accessibility. Interestingly, both types of lamin influence chromatin positioning near NPCs, indicating that lamins and nuclear pores jointly modulate genome organization [9,11]. This work demonstrates that cryo-ET – based visualization of the nucleus provides structural information within its native cellular context, thereby enabling functional insights into how the nuclear lamina integrates higher-order structural organization with genome organization in intact cells.

### Nuclear pore complexes

The nuclear pore complex (NPC) is one of the largest protein assemblies in the cell, mediating selective transport between the nucleus and the cytoplasm. Composed of ~30 nucleoporins arranged in multiple subcomplexes, the NPC has an estimated molecular mass of ~100 MDa [77]. Cryo-ET studies of isolated nuclear envelopes yielded detailed models of the symmetric scaffold [78,79] but failed to capture *in vivo* conformational variability.

Early studies of the NPC structure using intact nuclei and cells, provided modestly resolved structures with isotropic resolution [80]. Cryo-FIB-milling followed by cryo-ET allowed the reconstruction of NPCs from thin lamellae yielding higher resolution. The structure of NPCs from human, yeast, and algal cells revealed substantial differences between species and differences in diameter compared to purified samples [13,81–83]. In particular, the central channel diameter was found to be up to 75% larger in intact cells than previously estimated. Such differences highlight the influence of the native cellular environment on NPC conformation and emphasize the importance of *in situ* approaches. Further cryo-ET studies demonstrated that NPC architecture is dynamic and mechanosensitive. In yeast, the NPC scaffold dilates during exponential growth but constricts under osmotic shock or energy depletion, halving the central channel volume [82]. These structural changes were reversible and correlated with nuclear volume shrinkage, suggesting that membrane tension in the nuclear envelope regulates NPC diameter. This mechanosensitivity provides a direct link between nuclear mechanics and nucleocytoplasmic transport and a structural interpretation of previous observations [84], with implications for processes such as cell migration and differentiation where variations in nuclear mechanics may modulate local pore diameter.

Another long structural feature of the NPC, the flexible nuclear basket, has recently been resolved *in situ* [85]. In yeast, eight filaments emanate from the nuclear face of the NPC, converging into a closed ring resembling a basket base. In mammalian cells, the basket appeared more flexible and open, lacking the distal connections observed in yeast, however, this may reflect structural flexibility rather than genuine loss of these elements. These differences point to evolutionary adaptations in nuclear transport machinery. Remarkably, baskets created exclusion zones that prevented chromatin from approaching NPCs, consistent with early hypotheses that pores organize gene expression by gating transcriptional activity [86,87] and recent cryo-ET chromatin visualization studies discussed above [9,66]. The dynamic behavior of basket nucleoporins suggests that NPCs may functionally specialize by adopting distinct conformations and molecular compositions. More recently, in situ cryo-ET combined with live imaging revealed that Ran-dependent transport gradients actively impose asymmetry on NPC peripheral nucleoporins and therefore to the regulation of nucleocytoplasmic transport [88].

Cryo-ET has also illuminated NPC turnover and viral nuclear import. Studies in yeast revealed NPC turnover, where NPC-containing herniae bud from the nuclear envelope before degradation by autophagy [89]. In HIV-1 infection, correlative cryo-ET demonstrated that intact cone-shaped viral capsids, ~60 nm in width, could traverse dilated NPCs, resolving a paradox posed by earlier models in which NPC channels were thought narrow [90]. However, this appears to be the case only for *in situ* reconstituted NPCs, as earlier structural studies using isolated nuclei and nuclear membrane fail to showcase the dynamic nature of *in cellulo* structures. Beyond modest changes in NPC diameter, subsequent work reported pronounced pore deformation during the viral core entry [91,92]. Consistently, both mechanical remodeling of the NPC and the intrinsic mechanical properties of the capsid modulate import efficiency, supporting a model in which the capsid undergoes force-dependent constriction while translocating through the pore [92]. These findings exemplify cryo-ET’s ability to capture dynamic cellular events by visualizing structural snapshots directly in situ. The preservation of native cellular context together with high-resolution structural information proved crucial for understanding functional aspects of the nuclear envelope.

### Ribosome biogenesis and nucleolar organization

The nucleolus is a phase separated membrane-less organelle specialized in ribosome biogenesis. Single-particle cryo-EM has provided atomic structures of individual pre-ribosomal intermediates, but these studies required purified samples and often missed transient or context-dependent interactions [93]. Cryo-ET allows visualization of the nucleolus in its native state, revealing how ribosomal precursors are spatially organized during maturation [94].

Tomographic analyses showed that ribosomal precursors are organized in gradients, with particle size and maturation increasing radially from the nucleolar center to the periphery [94]. Small subunit processomes, serving as construction scaffolds, were confined to nucleolar regions, while more mature complexes localized toward the nucleoplasm. These findings provide direct molecular evidence for models proposing that nucleolar compartmentalization emerges from concentration gradients and multivalent interactions, consistent with liquid – liquid phase separation principles. Importantly, cryo-ET identified densities and cofactors absent from purified preparations, highlighting once again the importance of studying nuclear processes in situ.

## Perspectives and future directions

High-resolution imaging has transformed our understanding of nuclear and cellular organization. Cryo-ET is reshaping nuclear cell biology by enabling molecular-scale reconstructions of nuclear assemblies within their native context-rich environment. The studies highlighted above – spanning chromatin heterogeneity, nuclear pore complex architecture and mechanosensitivity, nucleolar gradients, and lamin – chromatin coupling – offer unprecedented insight into the dynamic structural logic of the nucleus. As cryo-ET methods continue to evolve, they are poised to enable further major discoveries in nuclear organization and function. Increased throughput and improved signal-to-noise are expected to broaden its impact, enabling investigation of processes such as nuclear condensate formation, principles of three-dimensional chromatin organization during development and cell division, mechanisms of nuclear rupture and repair, and viral nuclear entry and genome integration. In mechanobiology, molecularly resolved structural information offers a powerful framework to link force-induced conformational changes to cellular function, as exemplified by recent work on nuclear pore complexes.

Nevertheless, the full potential of cryo-ET has yet to be realized. The inherently low signal-to-noise ratio of single tomograms currently limits the level of detail that can be extracted from individual datasets. Improving the resolution of single tomograms therefore remains a central technical challenge, since cellular scenes are unique and may not enable averaging procedures. Recent technological developments – including laser phase plates [95] and renewed exploration of helium-temperature cryo-EM [96] —may enhance contrast and preserve high-resolution information. In parallel, advances in direct electron detectors, automated acquisition pipelines, and higher-throughput imaging workflows are steadily increasing data quality and efficiency. Complementary progress in correlative and integrative imaging approaches, such as cryo-CLEM, expansion microscopy, and time-resolved cryo-ET, further extends the interpretive framework by linking ultrastructure with spatial and temporal cellular context [97,98]. Moreover, innovations in vitrification strategies, expansion of samples that can be processed and imaged [43,46], optimized substrates for adherent cells, and controlled mechanochemical perturbations prior to freezing promise to broaden the physiological relevance and experimental versatility of cellular cryo-ET.

Alongside these hardware and acquisition advances, rapid progress in computational analysis is redefining what can be extracted from inherently noisy cellular datasets. Because many nuclear assemblies occur in low copy numbers or exist only transiently, subtomogram averaging or template matching are not always applicable, particularly within the dense nuclear environment. Emerging deep-learning strategies for denoising, segmentation, and particle identification are beginning to overcome these limitations, enabling the detection of sparsely distributed or short-lived macromolecular complexes directly within individual tomograms [57,59–61,99,100]. These developments move the field closer to the long-standing vision of visual proteomics [99,101].

Together, these developments position cryo-ET at the forefront of visualizing molecular organization, allowing previously unattainable insights into the structures that shape cellular function.

## Data Availability

This article is a review of previously published literature. No new data was analyzed in support of this article.

## Dictionary

**Template matching**: Template matching is a computational strategy in which a predefined structural reference (template) is scanned across the tomogram/dataset to identify similar features.

**Subtomogram averaging (STA)**: is the computational alignment and averaging of several copies of a macromolecular structure to enhance signal-to-noise and reveal higher-resolution structural details.

**Back-mapping**: the process of aligning a refined subtomogram average volume back into its original position and orientation within the tomogram or cellular volume.
